# Conceptual design of smart multi-farm produce dehydrator using a low-cost programmable logic controller and raspberry pi

**DOI:** 10.12688/f1000research.54463.1

**Published:** 2021-08-16

**Authors:** Sunkanmi Oluwaleye, Victoria Oguntosin, Francis Idachaba

**Affiliations:** 1 Electrical and Information Engineering Department, Covenant University, Ota, Ogun State, Nigeria; 2 KMF Concept Limited, Egbeda, Lagos State, Nigeria

**Keywords:** Dehydrator, Low-Cost PLC, Multi-Farm Produce, Raspberry Pi

## Abstract

**Background:** Acceptable food processing techniques require the removal of water contents from the crop or food sample without destroying the nutritional qualities of the food sample. This poses a strict requirement on the dehydrator or oven that will be used in the dehydrating techniques to have the ability to control both temperature and humidity of its drying chamber.

**Methods**: This work centres on how an autonomous multi-farm produce dehydrator that can also serve as an oven can be designed with a raspberry pi and a low-cost programmable logic controller (PLC). The dehydrator gives the users the flexibility to control both the drying chamber’s temperature and humidity from its web interface via a mobile device or the dehydrator’s HMI. Heat energy from the Liquid Petroleum Gas (LPG) is used so that the dehydrator can be readily available for commercial or industrial use.  The small electricity required to power the electronics devices is obtained from the hybrid power solution with an electric energy source from either the mains electricity supply or solar..

The design was tested by creating an operation profile from the proposed web application for the dehydrator. The operation trend was analysed from the web application’s Trendlines page.

**Results:** The report showed that both the temperature and humidity of the dehydrator could be controlled, and access to historical operation data will give insight to the user on how to create a better operation profile.

**Conclusion:** The setup described in this work, when implemented was able to produce a dehydrator/oven whose temperature and humidity can be perfectly controlled and its generated heat is evenly distributed in its drying chamber to ensure efficient and effective drying techniques use in crop preservation and food processing.

## Introduction

Over the years, the need for postharvest food preservation has been on the high increase, especially in Africa, where farmers depend on the natural climate for the cultivation of their crops. For this reason, the harvest of crops has been seasonal, leading to a surplus of farm produce at one time, hence, postharvest wastage, and scarcity at another time. ^1^ Furthermore, things have become worse with the recent inconsistency in the climate, which has negatively affected the farmers’ productivity. ^2^

Therefore, it has become very critical to optimise the farmers’ harvest using postharvest preservation technique with dehydrators that are built for African farmers and food processors. ^3^ An ideal dehydrator removes the water content from food crops using heat and airflow in a controlled environment while not compromising the nutrient quality of the crops. ^4^ Several studies have been done on the development of crop dehydrators. ^5- 8^ The use of electricity was used in the design and fabrication of portable dehydrator in Nadu’s study, ^5^ where the dehydrator chamber’s temperature and airflow were controlled with the use of heating coils and an air blower, respectively. Also, the use of renewable heat energy from the sun was used as described by R. O Lamidi, ^6^ where the harvested heat energy was distributed into the drying chamber with the help of an air blower. This approach was extended to the design of a hybrid dehydrator where liquid petroleum gas (LPG) was used to generate the required heat energy when solar energy was not available.

**Figure 1.**
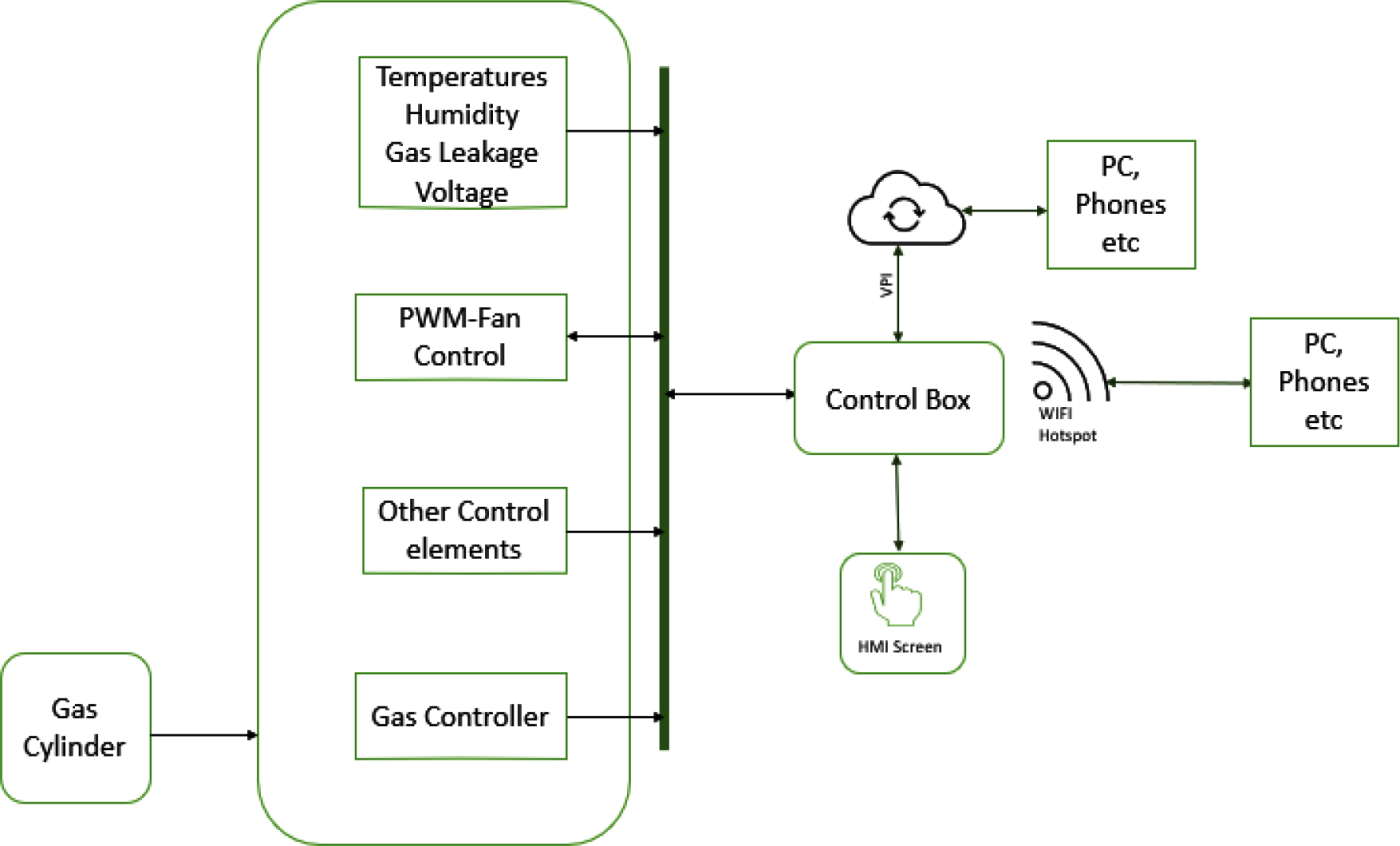
Block diagram of proposed dehydrator.

This work aims to design an autonomous multi-farm produce dehydrator that can also be used as an oven. Unlike the other works cited above, the dehydrator will be used for industrial purposes; the user can create his or her operation profile for a dehydrating process via the dehydrator’s web interface. The operation profile is stored by the device and can be used for future drying operations. As shown in Figure 1, a user can monitor the drying process or download previous operation data using the local web interface of the dehydrator or from its small human-machine interface (HMI).

The work will focus on the conceptual design of the dehydrator from the mechanical structure to the electrical components, including the software development. A low-cost programmable logic controller (PLC) and Raspberry Pi will be used to create industrial-grade electronic hardware for the dehydrator; the user will be able to create an operation profile for a particular type of crop by setting the target temperature, target humidity, maximum fan speed, minimum fan speed, and duration for the operation. The proportional–integral–derivative (PID) control program running on the PLC will use a feedback control loop mechanism to ensure the drying operation is carried out as specified in the configured profile. ^9^ The raspberry pi functions as a computer to handle remote connectivity and local resource management such as data storage and hosting of the web application responsible for the system operation. Finally, the user will be able to download the previous operation data from the system for further analysis.

### Literature review

Over the years, the drying of crops has been advancing using continuously improved techniques to attain the goal of safely removing desired moisture content from crops without compromising their nutritional qualities. ^7, 10^ Drying methods can be broadly categorised as natural drying and artificial drying. ^4^ Natural drying makes use of direct heat from the sun for drying; the sample is spread on the horizontal plane that is opened to direct sunshine without any shade and heat energy from the sun is used in the drying process. This is regarded as passive solar drying. ^11^ The problem with this approach is that the drying process is dependent on environmental conditions, which posed a serious limitation on the sample drying rate. Unfortunately, during the harvest period of most crops in both tropical and subtropical zones, the environmental conditions are usually not favourable because of the rainfall. Hence, the need for an artificial drying method becomes paramount. Artificial drying techniques can be categorised as: hot air convection drying, freeze and vacuum drying, drum drying, and spray drying.

Hot air convection drying is the most commonly used artificial drying technique because of its relatively low cost of production and flexibility. It can be set up mechanically without the use of electricity, complex structure, and electronic control, making it suitable for farmers in a very remote area who need to dry for personal storage or small-scale business demand. In this scenario, the source of heat energy can either be biomass, geothermal, waste heat, oil, natural gas, or solar. ^11- 13^ Sometimes these sources are combined as a hybrid heat source. ^14, 15^ The problem with this approach is that the drying process compromises the nutritional quality of the product because the drying conditions (temperature, humidity, and air velocity) cannot be adequately controlled. To solve this problem, C. Pacco ^8^ demonstrated how temperature control could be simulated in a dehydrator using LabView software version 2016; this can be implemented in hot air convection drying where the heat energy source, air velocity, and humidity can be controlled. Automated hot air convection drying can broadly be categorised as static or discontinuous drying, and continuous drying. Static or discontinuous drying is used for small or medium-scale food drying where complex structure and control techniques can be afforded. ^16^ This comes in the form of batch tray drying, where the sample has to be dried batch by batch. The samples are stacked stationarily in the drying chamber. The major problem with this approach is that the samples closer to the heat source are dried faster, causing non-uniform drying of the whole drying samples. Because of the cost-effectiveness of this drying approach, the goal of this work is to use this static drying and, at the same time, evenly distribute the heat across the drying chamber in an efficient and uniform manner.

Continuous drying with hot air convection requires a more complex structure and more robust industrial control to meet the need of large-scale industrial food drying. Uniform drying is easily achieved in the continuous dryer because the mass drying sample is in a continuous circulation within the drying chamber until the drying time is reached. Examples of this type of dryers are rotatory, tunnel, belt, fluidised bed, and impingement dryers. ^17^

Freeze and vacuum drying are techniques of drying that do not involve heat. ^18^ Because heat is not involved, the product dried usually retains their nutritional qualities. The downside to this approach is cost, especially for the small and medium scale crops and food processors. The drum dryer is used for drying paste or slurry roll over a heated drum ^19^ and the drying time is determined by the drum’s rotational speed. It is very popular with the pharmaceutical industries. Spray dryers are advanced application-specific dryers. Through the spray drying process, dried powders are produced from the liquid slurry or paste, ^20^ which procudes products such as powdered milk.

The goal of this work is to optimise a tray-type hot air convection dryer. As demonstrated by M. S. Badahman and Y. S. Susiapan, ^21^ a NodeMCU ESP8266 main microcontroller was used to build a smart oven that estimated the drying/cooking time of a particular food product by processing the weight of the food obtained by integrating loadcell into the system. A thermocouple was used as a temperature sensor; it senses the temperature of the oven’s drying chamber, and based on the temperature value, the microcontroller either turns on or off the electric heater that generates the required heat energy for the oven. Because a NodeMCU ESP8266 was used, it can be easily integrated into the cloud for remote monitoring. The limitation of this approach was that no provision was made for heat distribution and hot air velocity control. This means that the approach will not be suitable for a bigger oven that requires uniform drying. In this work, the dryer’s drying chamber humidity with be controlled by varying the speed of the extractor fan that moves out the moist air from the system. Also, a Proportional, Integral, and Derivative (PID) temperature control will be used to ensure that the samples are dried with even temperature control for better quality.

In S. Istiqphara and N. Adliani’s study, ^22^ an adaptive dryer was developed for drying medicinal plants using the heat energy generated from the solar collector. The main controller used was a microcontroller connected to a DHT11 temperature sensor that captured the temperature of the drying chamber. The temperature of the drying chamber is regulated using two extractor fans. These fans come on to remove the excess heat in the drying chamber to maintain a constant temperature. A fuzzy logic PID controller is used to control when the fans come on and how long they have to stay on. The internet of things (IoT) was implemented by using a raspberry pi minicomputer that was connected to the microcontroller and the remote server over the internet. With this, end-users can connect to the dehydrator with their laptops or mobile devices to monitor the drying operation. However, the humidity of the drying product is not considered in this work which will limit the use of the dehydrator to drying products that are not affected by the moisture content of the drying air. In this work, the user will control the system humidity and the temperature simultaneously to ensure effective and qualitative drying, as shown by S. Misha *et al*. ^23^ Furthermore, S. Istiqphara and N. Adliani’s ^22^ system may not be commercially feasible when a high production rate of dried samples is required.

In a nutshell, several works have been done to improve dehydrator/oven operation performance by coming up with different techniques to control the drying temperature and humidity, as stated above. However, none of these designs have single-handedly control of both the temperature and humidity of the drying chamber simultaneously. In this work, the designed dehydrator will ensure the even distribution of heat within the drying chamber and, at the same, regulates both the temperature and the humidity of the drying chamber. Furthermore, the dehydrator will be user friendly, such that the user can create different operation profiles for different types of crops or any drying sample, and also set the desired duration for the operation.

## Methods

### Design

This paper focuses on a conceptual design of a dehydrator for drying multi-farm produce, the product has not been physically fabricated and tested. The mechanical or structural design has been carried out but not yet fabricated while the control or instrumentation design was done and tested on a physical PLC with sensors connected and on a raspberry pi board 3B+. The materials required to build this dehydrator can be categorised as mechanical or structural materials, and electrical or instrumentation materials. The structural parts include the drying chamber, heat exchanger, control box chamber, burner chamber, and the gas cylinder with its accessories. The heat energy generated for the drying operation is liquefied petroleum gas (LPG) because it is readily available and suitable for continuous industrial operation. The mechanical structure was designed using Autodesk Inventor Professional 2020 version 2020.1.1. The alternative open-source version that can be used is FreeCAD. The electrical/instrumentation parts consist of the gas controller, sensors, mini-PLC, raspberry pi, fans, HMI screen, AC/DC converter module, solar panel, solar charge controller, and the battery pack. The electrical/instrumentation parts will be described in greater detail later in this section. All these components are mechanically or electrically connected, as the case may be at the end of the component integration. The electrical components are powered from the 12Vdc source that can be autonomously backed up by the battery pack that is charged with AC/DC converter or the solar charge controller.

### Mechanical structure

The inner parts of the drying chamber are made of stainless steel to conform to the food safety standard. ^24^ The mechanical specifications of the dehydrator are shown in Table 1. The outer parts are made of iron steel plate to save cost. The space of 50mm between the inner plate and the outer plate of the drying chamber is loaded with insulation material such as fibreglass to prevent heat energy loss in the drying chamber as demonstrated by Brandon Tinianov. ^25^ The heat exchanger is installed inside the drying chamber, with its base protruding below the drying chamber into the burner chamber. The burner chamber houses the gas burner and the igniter. The gas supplied point on the burner is connected to the gas cylinder, whose opening is proportionally controlled by an electronic valve.

**Table 1.** The mechanical specifications of the dehydrator.

Dehydrator/oven attributes	Details
Dimension (mm)	710 × 610 × 1252
Weight (kg)	150
Drying tray dimension (mm)	300 × 400
Inner material	Stainless still
Drying parameter control	Temperature and humidity
Heat energy source	Gas (LPG)
Power source for electronics	230VAC or 12VDC solar power system
User Interface and connectivity	HMI Screen, local web interface that can be accessed with smart devices and/or a PC over a WiFi connection

**Figure 2.**
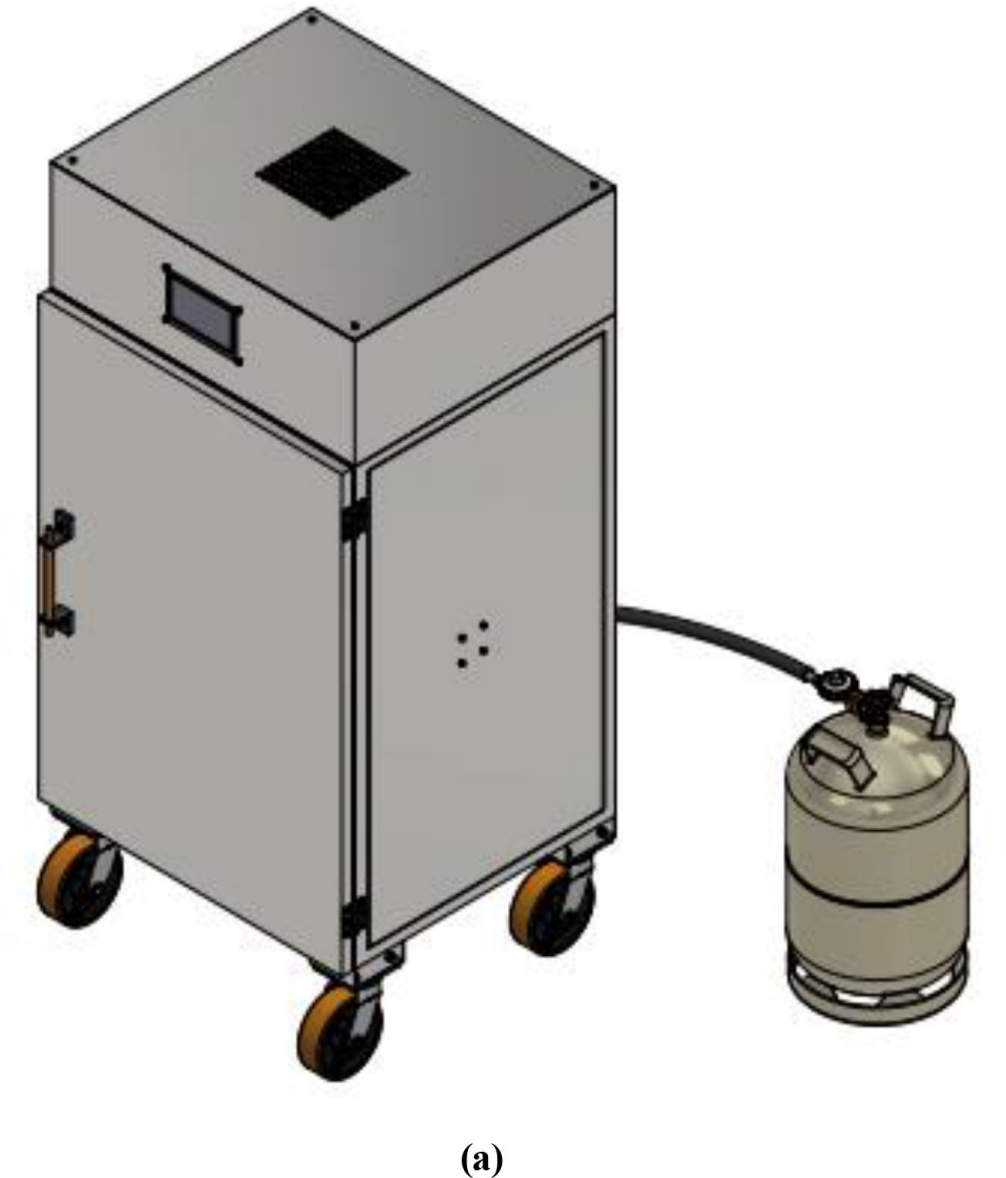

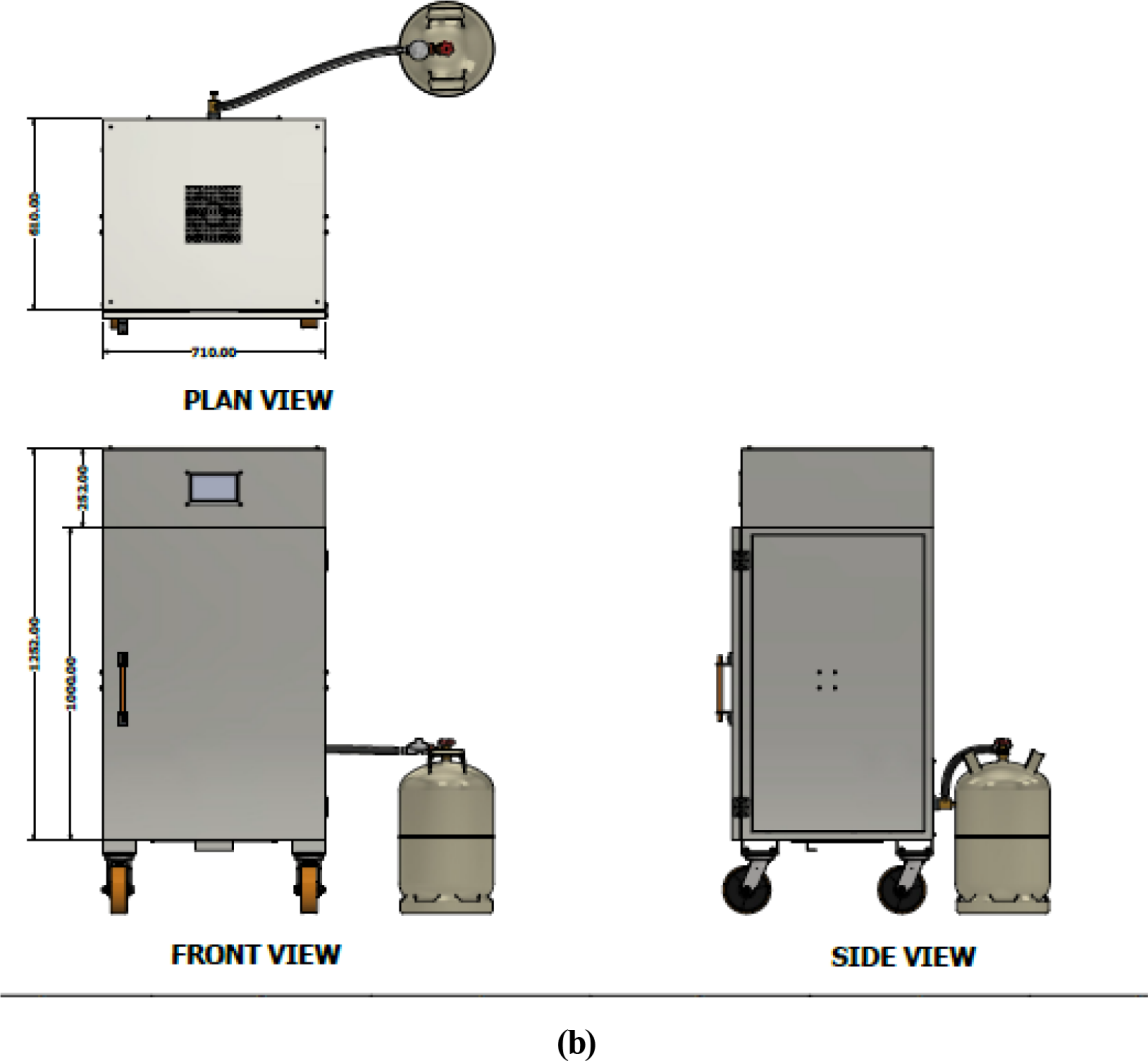
(a) Final outer structure of the dehydrator designed with AutoCAD inventor. (b) Dimensions of the oven in millimeters (mm).

**Figure 3.**
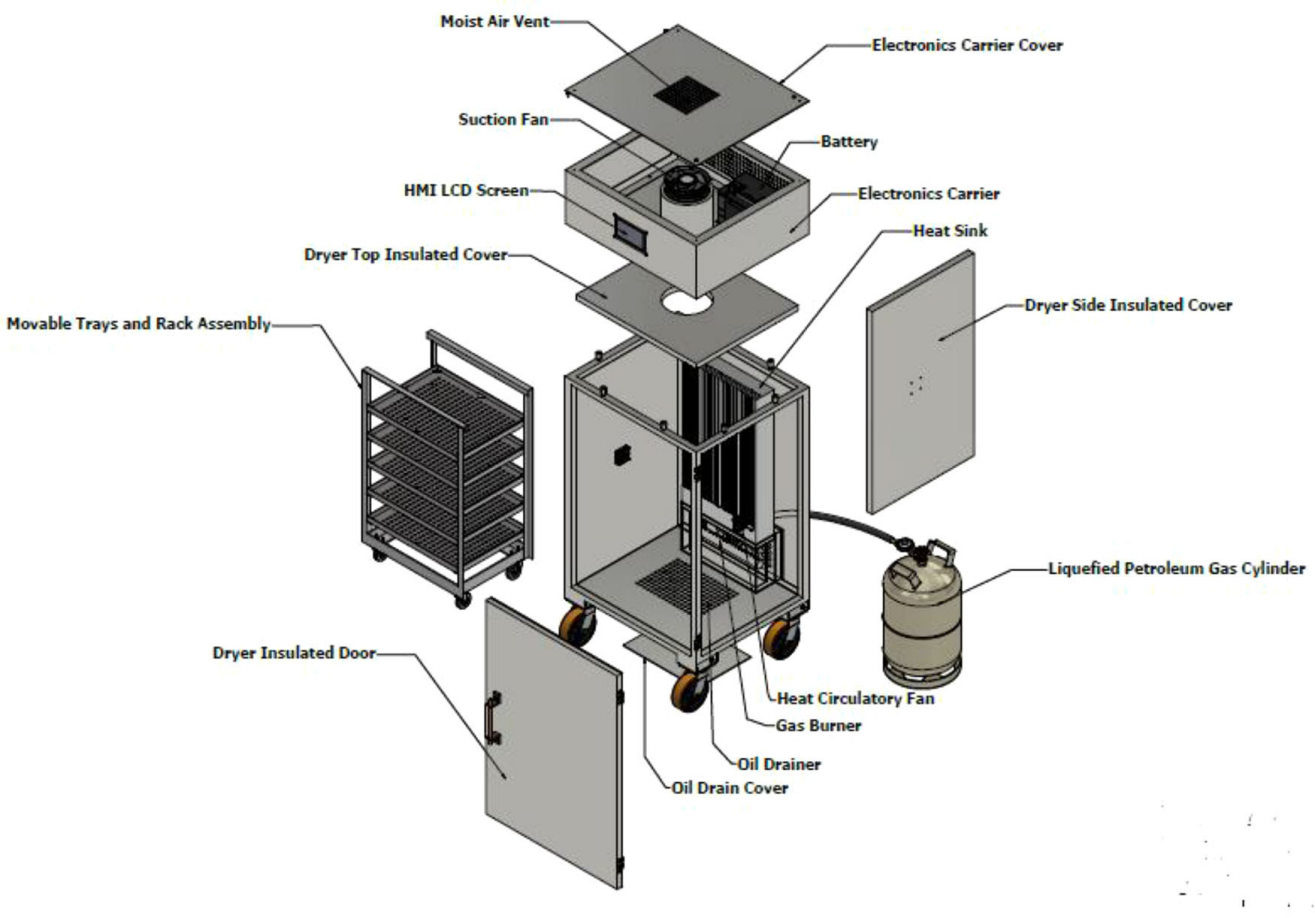
The main assembly of the dehydrator as exploded view including the movable tray, heat exchanger, electronic carrier, and other parts.

The control box is positioned on the top of the drying chamber; the box houses all the electrical components except the temperature sensors that are connected to the heat exchanger and the proportional electronic valve. At the centre of the control box is the cylindrical exhaust pipe that runs from the drying chamber to the top of the control box, where the exhaust fan is sitting. The exhaust cylinder is insulated to avoid damaging the electronics component with excessive heat and preventing heat loss from the system. The final structure of the dehydrator and the components of the dehydrator are shown in Figures 2 and 3.

### The electrical and instrumentation components

**Figure 4.**
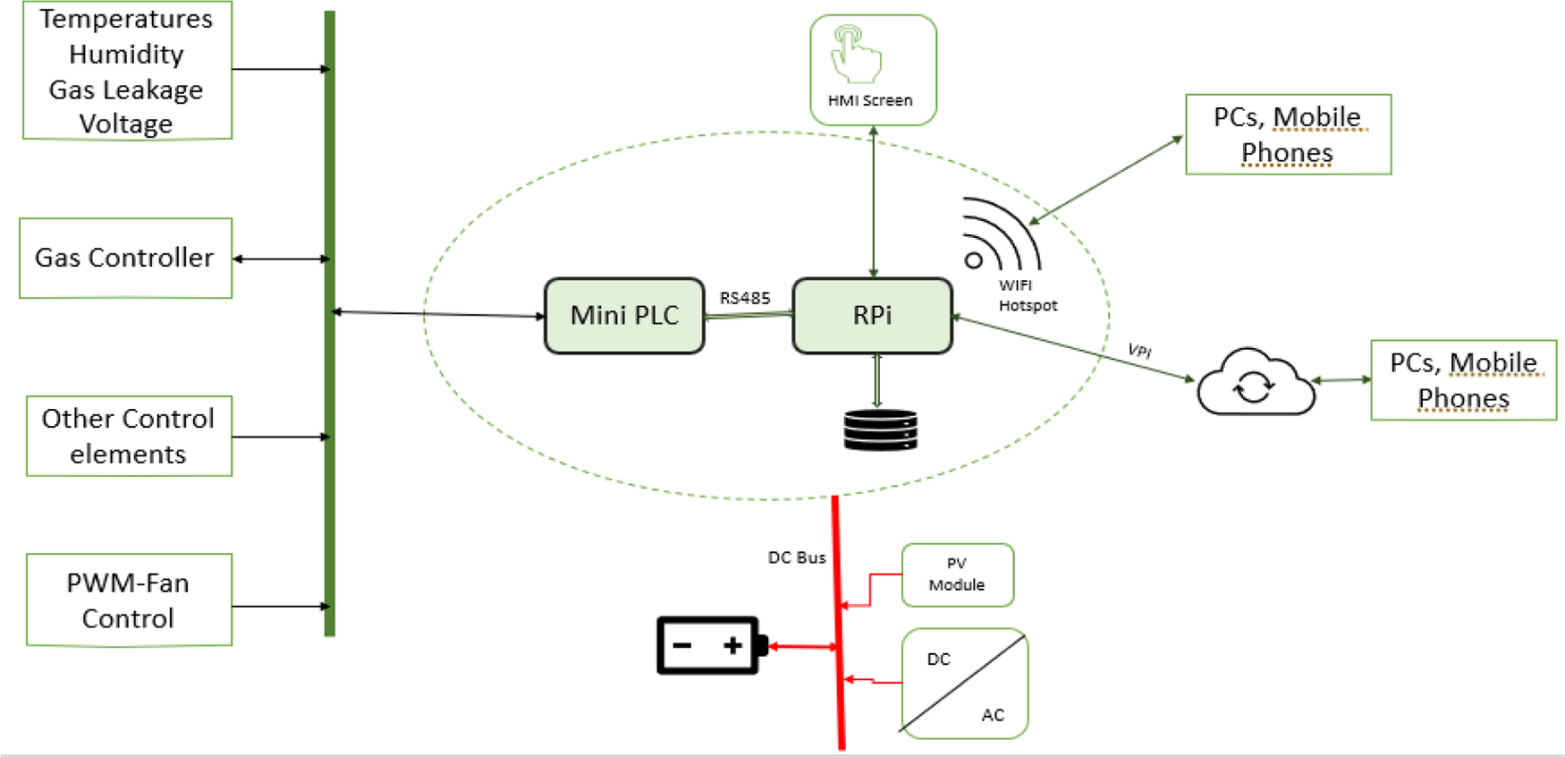
Electronical component architecture.

The components selected for this work are based on industrial-grade standards as recommended by the International Society of Automation (ISA) ^26^ and not from an experimental perspective. This is because this product is designed to be used in an industrial environment where an experimental board like Arduino would not be suitable as it is not protected against harsh, dirty, and electrically noisy environments. Furthermore, industrial actuators and electrical transducers cannot be easily integrated with Arduino without an extra interface board. Hence, using a PLC is a better choice because it is designed to operate in an industrial environment having inputs and outputs ports that can easily interface with industrial-grade sensors and actuators. The electronical component architecture is shown in Figure 4. The PLC handles the hardware interface (connections to all sensors and actuators) and control. At the same time, the raspberry pi plays the role of a computer, providing the web server that hosts the dehydrator web app, data storage, and supervises the PLC's operation. The user can interact with the system via its web interface over its Wi-Fi hotspot using a mobile device or PC. Alternatively, the web interface is also made available on the system HMI screen. The 12Vdc power source is provided from either the solar panel or the connected mains power, depending on which source is available.

### The mini-PLC

The mini-PLC is a low-cost version of the industrial programmable logic controller (PLC). PLCs are widely used in the industry because of its high processing speed and easy connection interface with industrial field devices such as sensors and actuators. ^27, 28^ The mini-PLC has a limited but sufficient number of inputs and outputs required for the work required in this design. The role of this PLC is to interface with all other hardware in the system.

**Figure 5.**
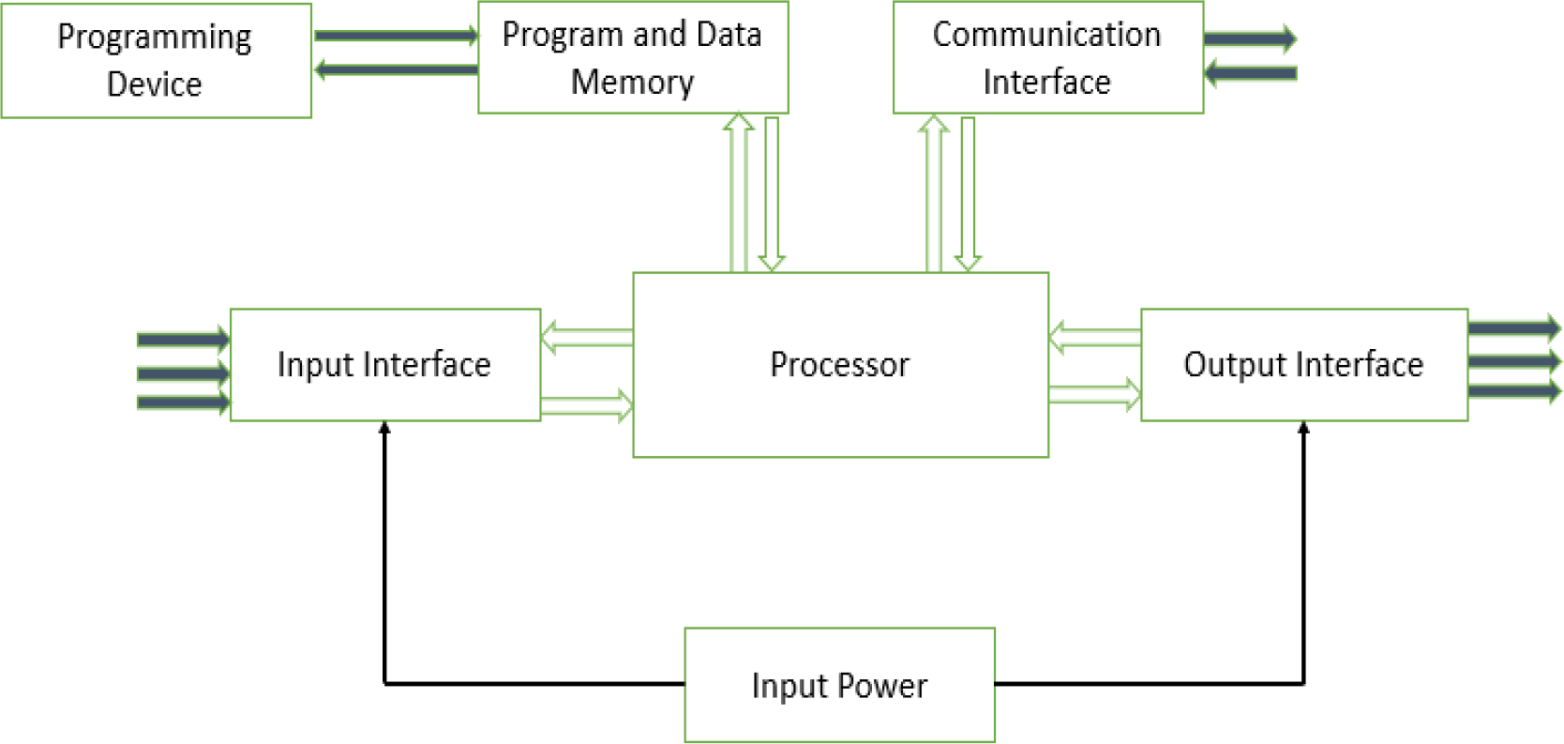
PLC basic structure.

The PLC basic structure is shown in Figure 5. The programming device, in most cases, is the personal computer (PC) on which the PLC programming software is running. In this project, GX Works2 Station version 1 was used, developed by Mitsubishi Corporation. From this software, the user can write the new software to the PLC and read the software that is already running on the PLC to the PC. The processor keeps cycling the code at a very fast speed at the same time, reading from the input register or bits and writing to the output register and bits as programmed in the running code.

**Figure 6.**
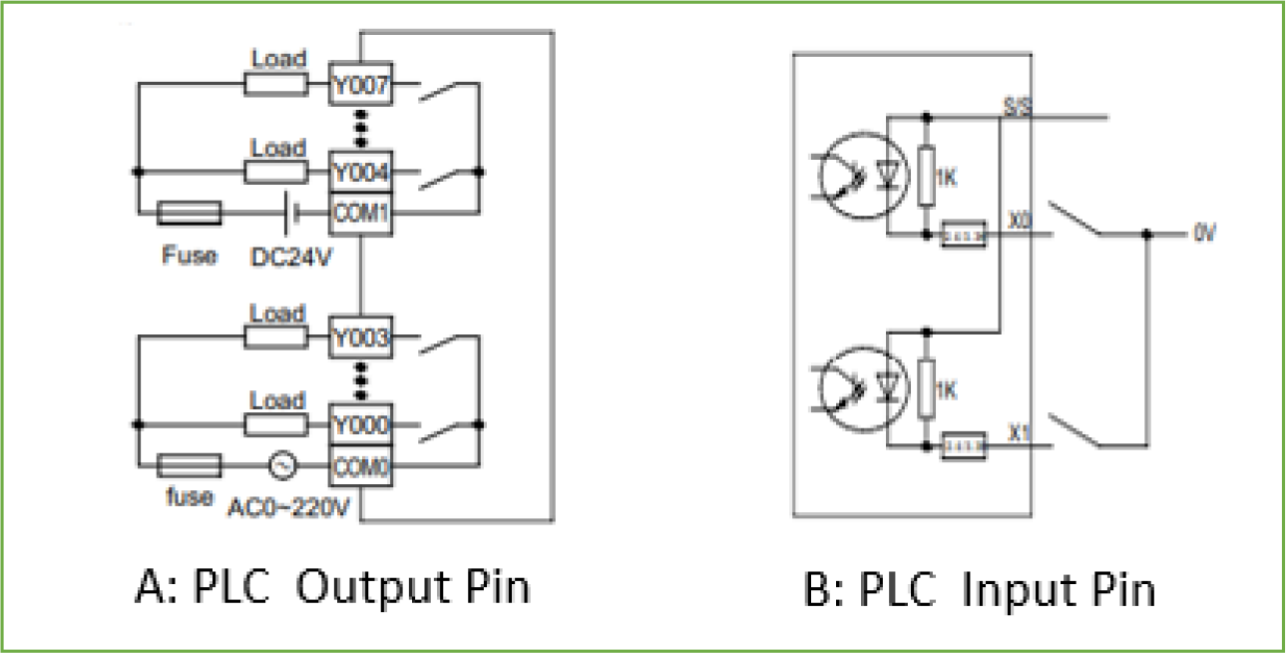
Electrical wiring of PLC input and output pins.

**Figure 7.**
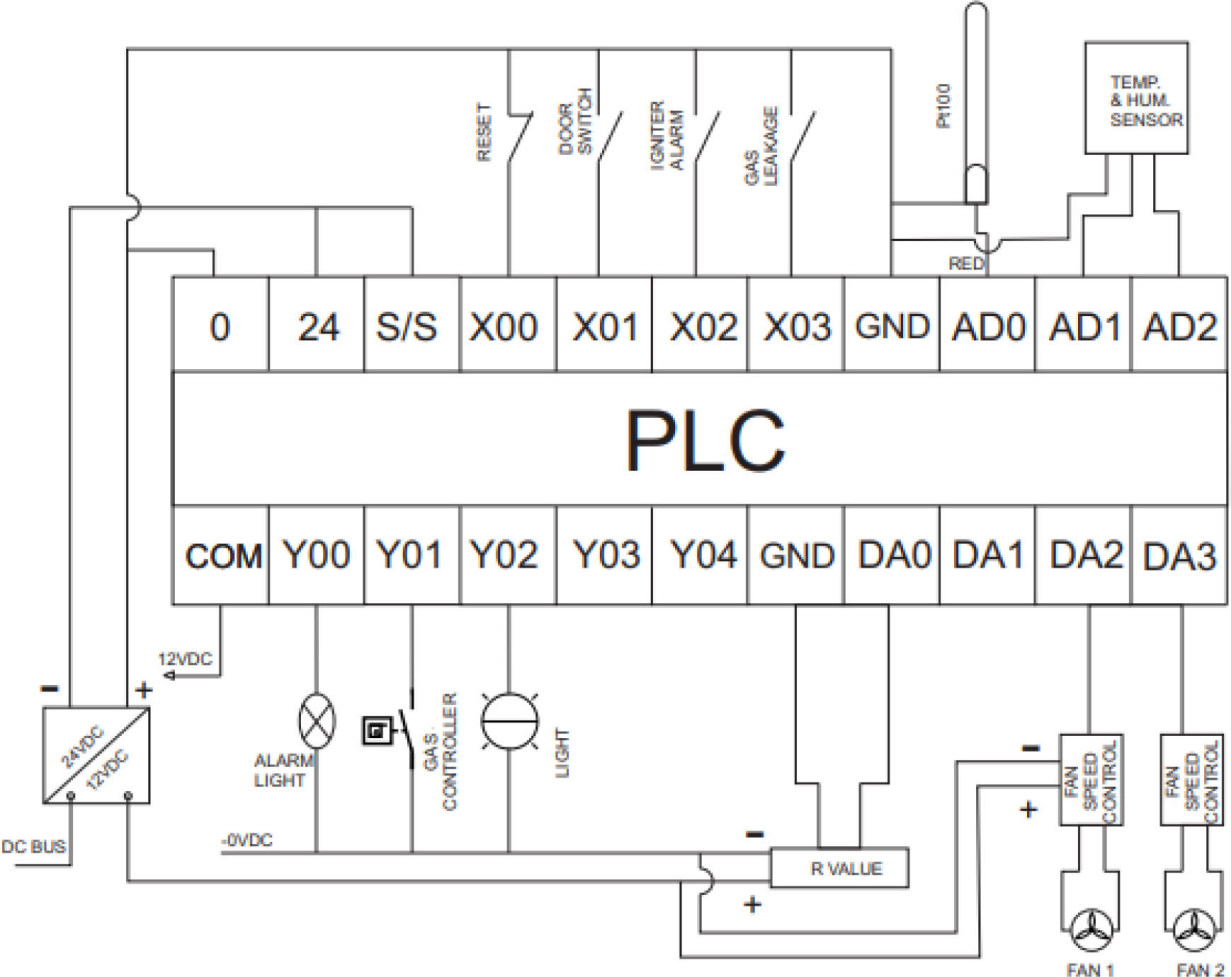
PLC wiring diagram.

The available input and output ports for the mini-PLC is given in Table 2. The acceptable wiring connection for the digital input and output port are described in Figure 6. The digital output is a dry contact relay type, and any voltage can be connected to the common pin of the digital output port. The Y output pin associated with the common pin is electrically linked when the output bit is activated within the PLC code. For the input pin X, the associated bit in the PLC code is activated if the input pin is grounded, as shown in Figure 6. The complete wiring diagram of the PLC is shown in Figure 7. The PLC is powered from a 12Vdc source, and the digital inputs signals trigger from the door switch, igniter alarm, gas leakage sensor, and reset button which are wired to the digital input pins of the PLC while the analogue input signals from the PT100 sensors and temperature and humidity sensors are wired to the analogue input pins of the PLC. The digital output actuators and analogue output actuator are connected to the PLC digital output pins and analogue output pin, respectively.

**Table 2.** PLC input and output ports.

Features	No of points	IO Types	Comments
Digital input	8	Passive NPN	Receives 24V GND as active signal
Analog input	4	one point of PT100 temperature sensor, three points of 4-20mA	12 bits ADC used
Digital output	8	Relay type	Normally open (NO) relay contact
Analog output	6	four points 0-10V signal, two points PWM 21kHz (3.3)	
Com ports	3	one RS232, two RS485	

### The minicomputer (raspberry pi)

The role of raspberry pi is to function as a computer, ^26^ and it will be constantly connected to the PLC using a Modbus RS485 communication protocol to get all operational data that will be stored on its MongoDB database. Furthermore, all user configurations will be set and stored on the raspberry pi. The settings can be done from the mini-HMI screen or the web interface built on the raspberry pi. ^25, 28^ Finally, the raspberry pi is the central control unit of the system; it decides when operation starts and ends and sets the operation parameter used by the PLC.

### The sensors

The list of the sensors used, and their function is given below:

➢ PT100 temperature sensors: three-wire type, used to obtain the temperature of the heat exchanger),
➢ Temperature and humidity sensor; with 4-20mA transmitter, used to obtain the temperature and the humidity of the drying chamber.
➢ Voltage sensor for the battery: The battery voltage sensor is a DC voltage transmitter that can measure up to 100VDC and transmit a 0-4mA output signal to the PLC. This is used to measure the battery voltage. The system intelligently uses this value to implement a battery low voltage disconnection process that prevents the battery from being over drained.
➢ Gas leakage sensor: This digital signal is triggered when there is gas leakage to ensure both the user and system's safety.
➢ Door switch: The system knows the status of the door with this input signal.
➢ Igniter alarm switch: The is helps the system to detect fault from the gas igniter controller.

All the sensors will be wired to the PLC, as shown in Figure 7.

### The gas ignition controller

The role of this device is to automatically ignite the gas burner in the burner chamber. It starts the ignition process based on the signal received from the PLC. It will afterwards send feedback to the PLC to confirm if the ignition process was successful or not.

### The power supply

This part comprises the 12V 100AH battery pack, the solar panel, the solar charge controller, and the AC/DC converter module. This part aims to supply the 12Vdc source required to power all the components in the system. The AC/DC converter can connect to a 230V AC source from the mains and gives 12Vdc output that can power the system and, at the same time, charge the battery pack. Also, if the mains power supply is not available, the solar panel can harvest solar power through the solar charge controller, which outputs 12Vdc that can power the system and charge the battery pack. The system power requirement is given in Table 3.

**Table 3.** System power requirement.

S/N	Component	Rated power (W)	Quantity	Consume power (W)
1	Fans	24	2	48
2	Proportional Valve	6	1	6
3	Shut Off Valve	6	1	6
4	Raspberry Pi 3 B	10	1	10
5	PLC	5	1	5
6	HMI	2.3	1	2.3
7	Light	5	1	5
Total power				**82.5**

The estimated time ( *T*_*b*_) for the battery to power the farm load at 45% depth of discharge (DoD) can be calculated as shown in equation 1:

$$T_{b}=\frac{\mathrm{DoD}\times B_{c}\times V_{s}}{L_{p}}.$$ (1)

*B*_*c*_ is the battery capacity, *V*_*s*_ is the system voltage, and *L*_*p*_ is the total power consumed by the system. Inputting the specific values of *B*_*c*_, *V*_*s*_, *L*_*p*_ and DoD gives the estimated time for battery power, *T*_*b*_ as shown in equation 2:

$$T_{b}=\frac{0.45\times 100\times 12}{82.5}=6.545454.$$ (2)

Hence, this means that the system can run on the connected battery for six hours and 32 minutes. This is based on the electrical parameters of this battery size as this has not been fabricated and tested.

A 150W 12V solar panel is used; the open-circuit voltage and short-circuit current of the solar panel are 18V and 9.4A, respectively. Two of these solar panels can be connected in parallel to charge the battery effective if the user wants to depend more on solar power. 30A 20V PWM solar charge controller is used to save cost; the MPPT version of this controller would have been better if the user will be comfortable with the price. A 230Vac input and 12Vdc out battery charger is used to charge the battery when the system is connected to the mains power.

### The software programming

**Figure 8.**
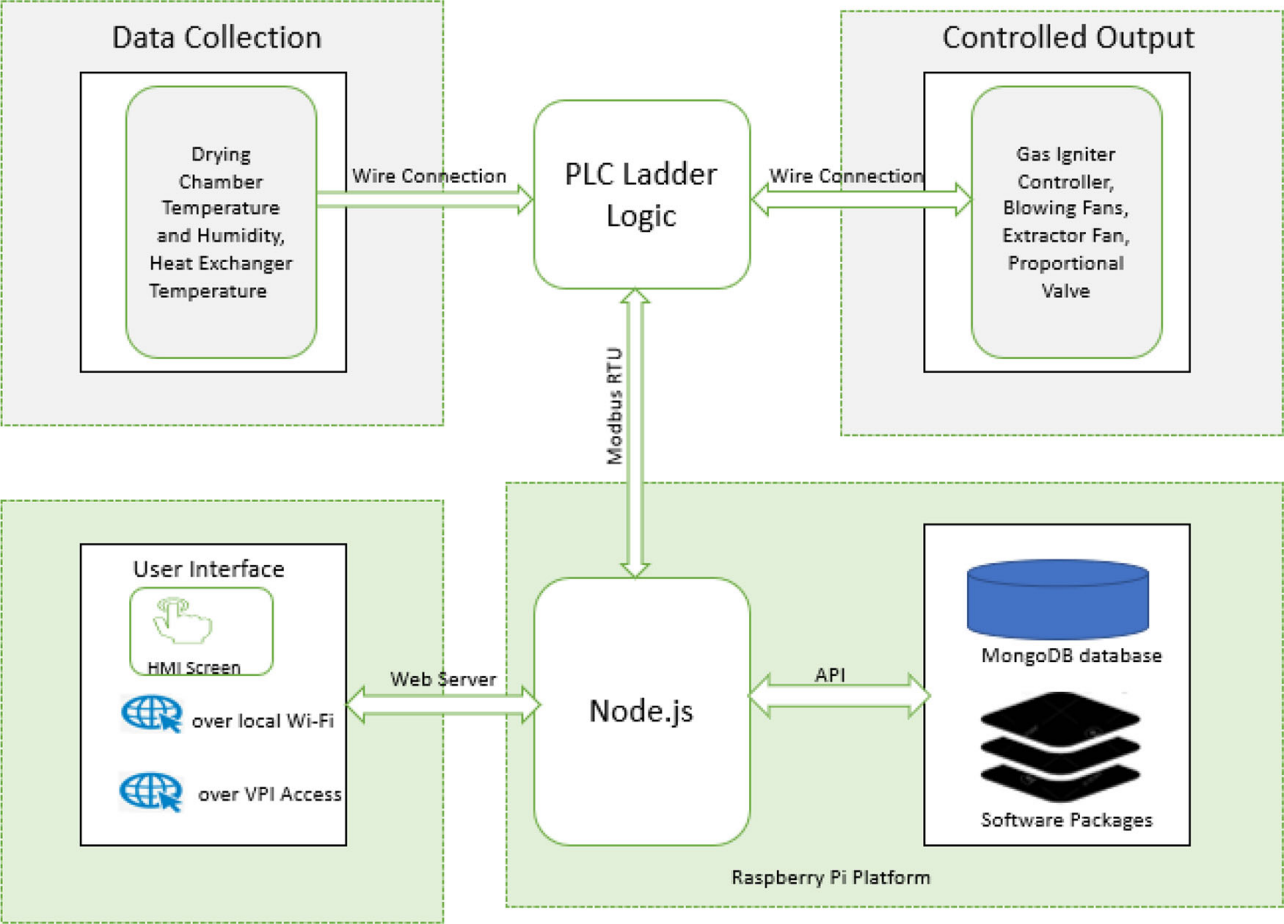
System software architecture.

The software development for this work will be categorised into two sections which are the PLC software program and the raspberry pi software program. Figure 8 shows the software architecture for this system. The web app that serves the users via the available web interfaces (the HMI screen and over the Wi-Fi connection) connects to the local server on the raspberry. JavaScript programming language was used to build both the frontend and the backend software. The backend software stores and retrieves data from the MongoDB server using mongoose API endpoints, and the backend software also communicates with the PLC using RS485 RTU protocol. The software on the PLC retrieves data from the raspberry pi via its Modbus assigned data registers and bits; these data determine the operation parameter such as target temperature, humidity, operation duration and alarm set points. Also, the PLC ladder logic software reads data from the connected sensors set the corresponding actuators accordingly. The details of the PLC software are further explained in the section below.

### The PLC ladder logic

**Figure 9.**
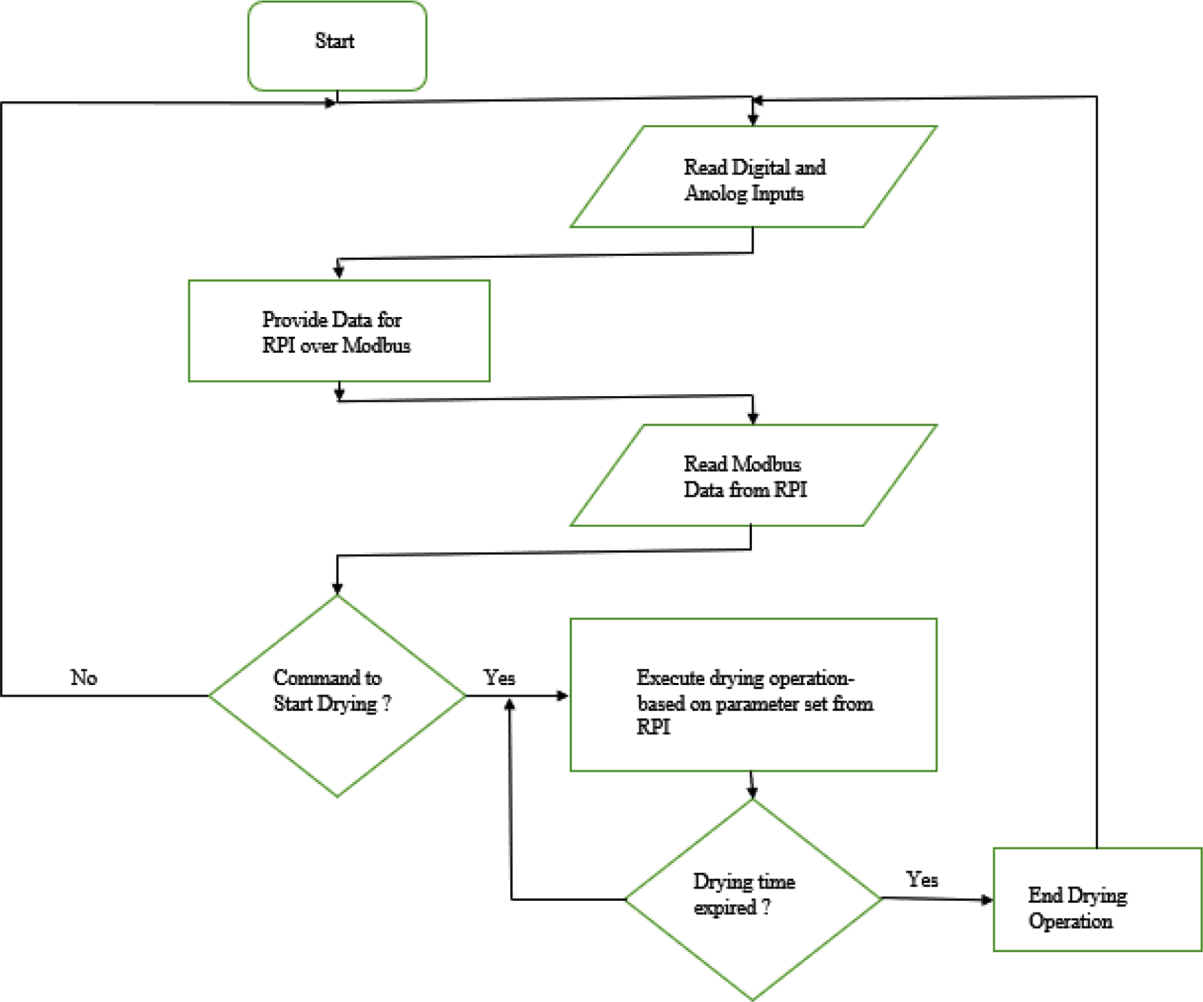
PLC program flow chart.

The ladder logic or ladder diagram (LD) is a programming language used for programming PLC in the automation industry. It is popular among automation engineers because it evolved from electrical relay circuits, making it easier to learn for anyone with basic knowledge of relay controls and electrical circuits. The primary functions of the PLC are carried out in the ladder logic program, as shown by the flow chart described in Figure 9. The PLC (a slave in the Modbus network) starts by reading the sensor data from the connected digital and analogue input sensors. These data are moved to the assigned Modbus data registers and bits to be read by the raspberry pi (the master in the Modbus network). The PLC also read Modbus data from the raspberry pi to get the command for the next action and obtain desired operation parameter from the user. The PLC executes dehydration operation based on the set operation parameter if the bit assigned for the command (‘start drying’) is true. The PLC keeps track of the operation time as desired by the user and detect if an alarm is activated.

### Reading analogue input with the PLC

The PLC has 12 bits ADC (Analogue to Digital Converter), and its analogue input values are read from data register D8030 to D8045; each register corresponds to the analogue input port AD0 to AD15. The calibration of this analogue input value is dependent on the characteristics of the sensor and the input type on the PLC. The analogue input type can be 0-10V, 4-20mA, k-type thermocouple, PT100, and others, depending on the application and the type of sensor that is selected. This PLC uses PT100 and 4-20mA input types. Table 4 indicates the individual sensors that are connected to the analogue input ports.

**Table 4.** PLC analogue input assignment.

Analogue input port	Data register	Connected sensor
AD0	D8030	Heat exchanger temperature
AD1	D8031	Drying chamber temperature
AD2	D8032	Drying chamber humidity

The calibration of both sensors used for drying chamber temperature and humidity is done by using the formula given below in equations 3 and 4:

$$y_{t}=\frac{x_{t}}{25}–40$$ (3)

$$y_{h}=\frac{x_{h}}{40}+1$$ (4)

**Figure 10.**
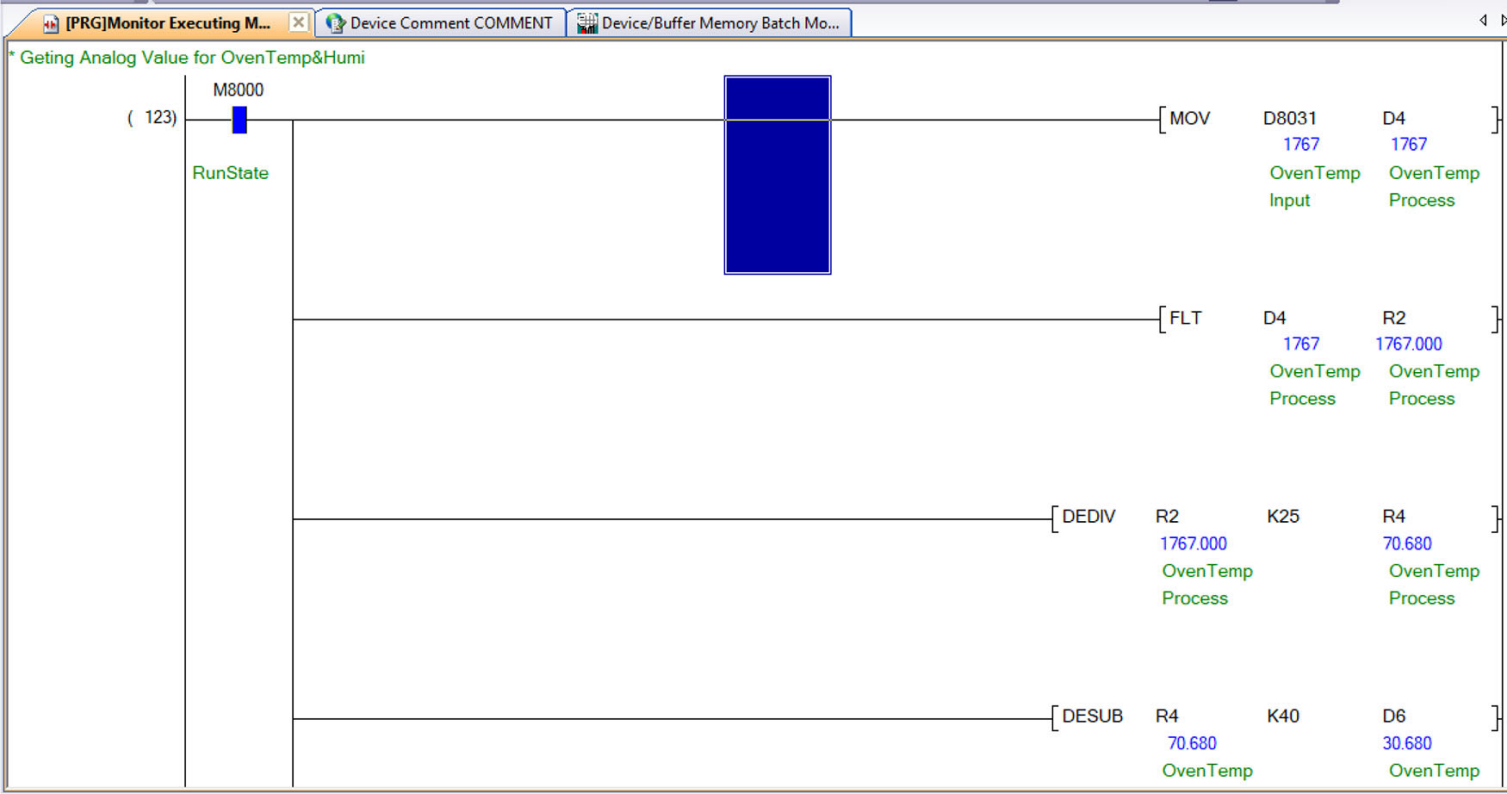
Temperature reading in ladder logic program.

*x*_*t*_ and *x*_h_ are the analogue values read from the data register for the connected temperature and humidity sensor, respectively. Similarly, *y*_*t*_ and *y*_*h*_ are the final temperature and humidity. Figure 10 shows how the dehydrator or oven temperature reading is obtained from the ladder logic program.

### PID control block in PLC

PID (Proportional Integral Derivative) control blocks are available in PLC. A PID controller is a control feedback mechanism used to regulate a plant’s process variable such as temperature, speed of an electric motor, pressure, flow rate, and other process variables. ^29^ The general model for a PID controller is given by equation 4:

$$C_{\mathrm{pid}}=K_{p}e\left(t\right)+K_{i}\int_{0}^{t}e\left(t\right)\mathrm{dt}+k_{d}\frac{\mathrm{de}\left(t\right)}{\mathrm{dt}}$$ (4)

where $C_{\mathrm{pid}}$ is the control signal for the system (PID output), *e*( *t*) is the error between the desired setpoint and the system output, *de*( *t*) is the derivation of error *e*( *t*), and $K_{i},K_{p}$ , and $k_{d}$ are integral gain, proportional gain and derivative gain, respectively.

Tunning of the PID means modifying the values of $K_{i},K_{p}$ , and $k_{d}$ accordingly. The role of the PID controller in this work is to regulate the dehydrator/oven temperature, humidity and fans’ speed.

**Figure 11.**
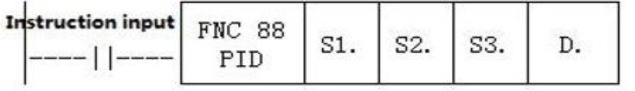
PID control block in the PLC.

To regulate the temperature, humidity, and the fan’s speed in this work, the command below is used to perform PID control that changes the output value according to the amount of change in the input. Setting the entries of Figure 11 based on the details of Table 5 activate a single input single output (SISO) PID function.

**Table 5.** PID block details.

Setting item		Content	Occupied points
S1.	Target value (SV)	Set target value (SV) PID instruction does not change the setting contents	1 point
S2.	Measured value (PV)	The input value of the PID operation	1 point
S3.	Parameter	Different parameter to set for desired functions and applications	25 points
D.	Output value (MV)		1 point

After setting the target value S1, the measured value S2, and the parameters S3~S3+6 in the execution program, the operation result (MV) is saved to the output value D. every sampling time S3.

### Raspberry pi software development

The two programming tools used to develop the raspberry pi software are Node.js linux binaries (ARM) version 14.17.3 and Linux Bash Shell Script. The shell script is used to automate the hardware configuration of raspberry pi on boot up, such as modem connection, activation of Nginx server, Wi-Fi hotspot setup, Virtual Private Internet Service activation and main program loading. The Node.js programming package is used to develop the main application, whose features are listed below:

➢ Frontend web interface: This provides a user-friendly interface for the dehydrator. The users can access the interface from the HMI screen or via their mobile devices or PCs over the dehydrator’s Wi-Fi connection using available web browsers. The user can view the current operating parameters of the dehydrator, download historical operation data, create an operation profile, and start/stop the operation process.
➢ Database Management: The application uses the MongoDB database, which is accessed using mongoose API endpoints. The current operation parameters are stored in the database only when an operation is active to conserve memory space. A newly created or edited operation profile by the user is also stored in the database.
➢ PLC operation control: The application connects to the PLC as a Modbus master to read data and set the PLC operation parameters.

### Software development on raspberry pi using Node.js

**Figure 12.**
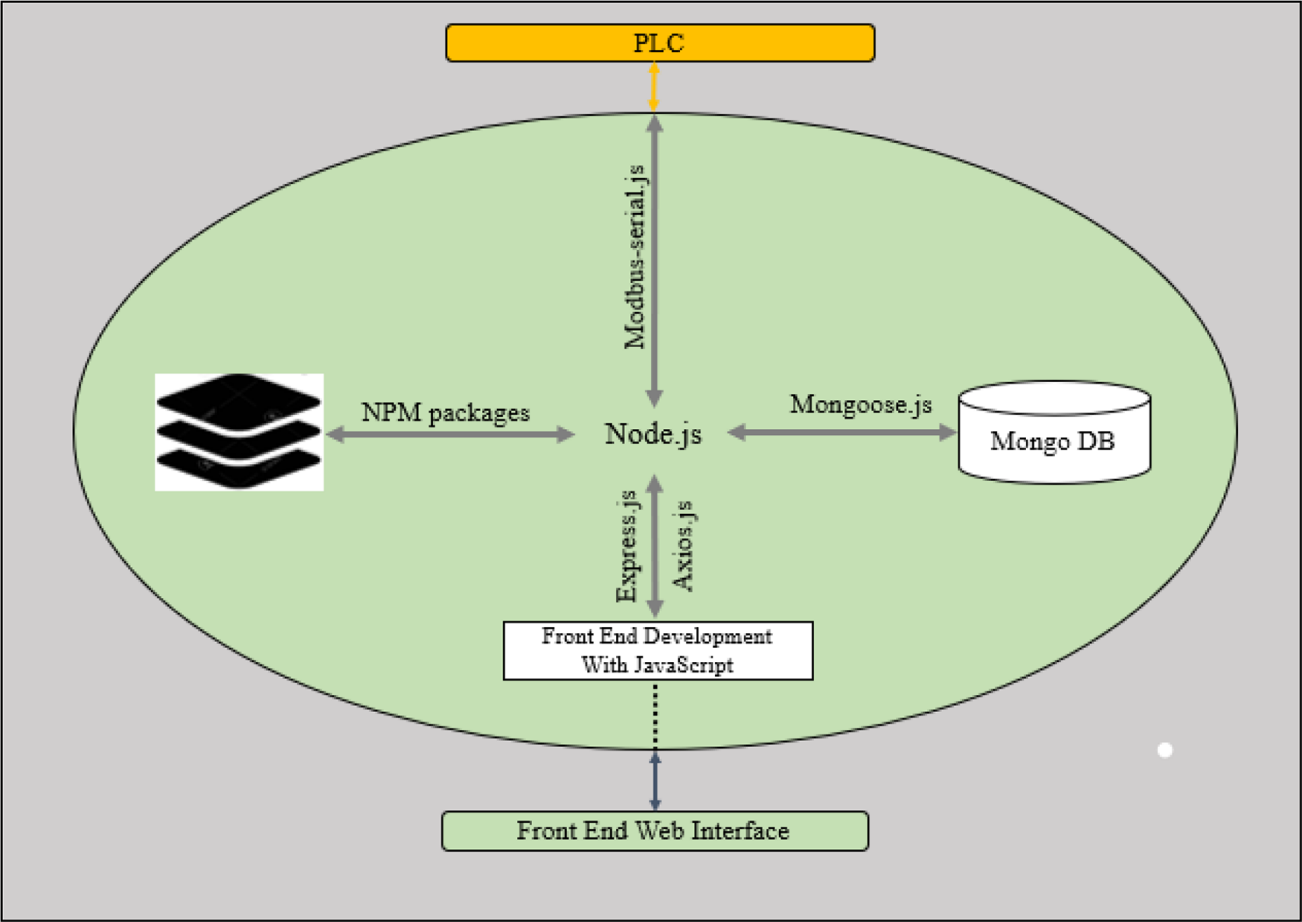
Node.js Program Structure on Raspberry Pi.

Node.js is a JavaScript runtime built on Chrome’s V8 JavaScript engine. V8 compiles JavaScript directly to native machine code using just-in-time compilation. ^30^ This approach makes it possible to write JavaScript language on the webserver side of our application and control the microcontroller hardware. Figure 12 shows the details of the Node.js App structure. The software development takes advantage of Node.js’s heavy support for libraries contributed by its community. Node package manager (NPM) helps to manage the node application’s dependencies on libraries used. The details of the major NPM packages in the application are listed below:

➢ Express.js: A node.js web application framework, this handles all HTTP request required by the application.
➢ Mongoose.js: This provides useful API endpoints to interact with the database from the node.js application. It is used to store created operation profile by the user, operation data read from the PLC and the activated alarms during the operation. It is also used to retrieve this stored data as required.
➢ Modbus-serial.js: It is used to implement RS485 Modbus RTU on the application. As a master on the Modbus network, the application can read and write data to the PLC that is acting as a slave on the Modbus network.
➢ Axios.js: This is used from the frontend to handle API request.

## Results

As this is a conceptual design, an operation test was performed to simulate how the system would perform in an enclosed chamber. A workbench test was carried out on how the temperature PID control will perform by creating an operation profile. In addition, the logs for this operation was downloaded and analysed to test the performance of the setup. The basic functionality of the frontend section was tested with both PC and Mobile phone.

### Operation test

**Figure 13.**
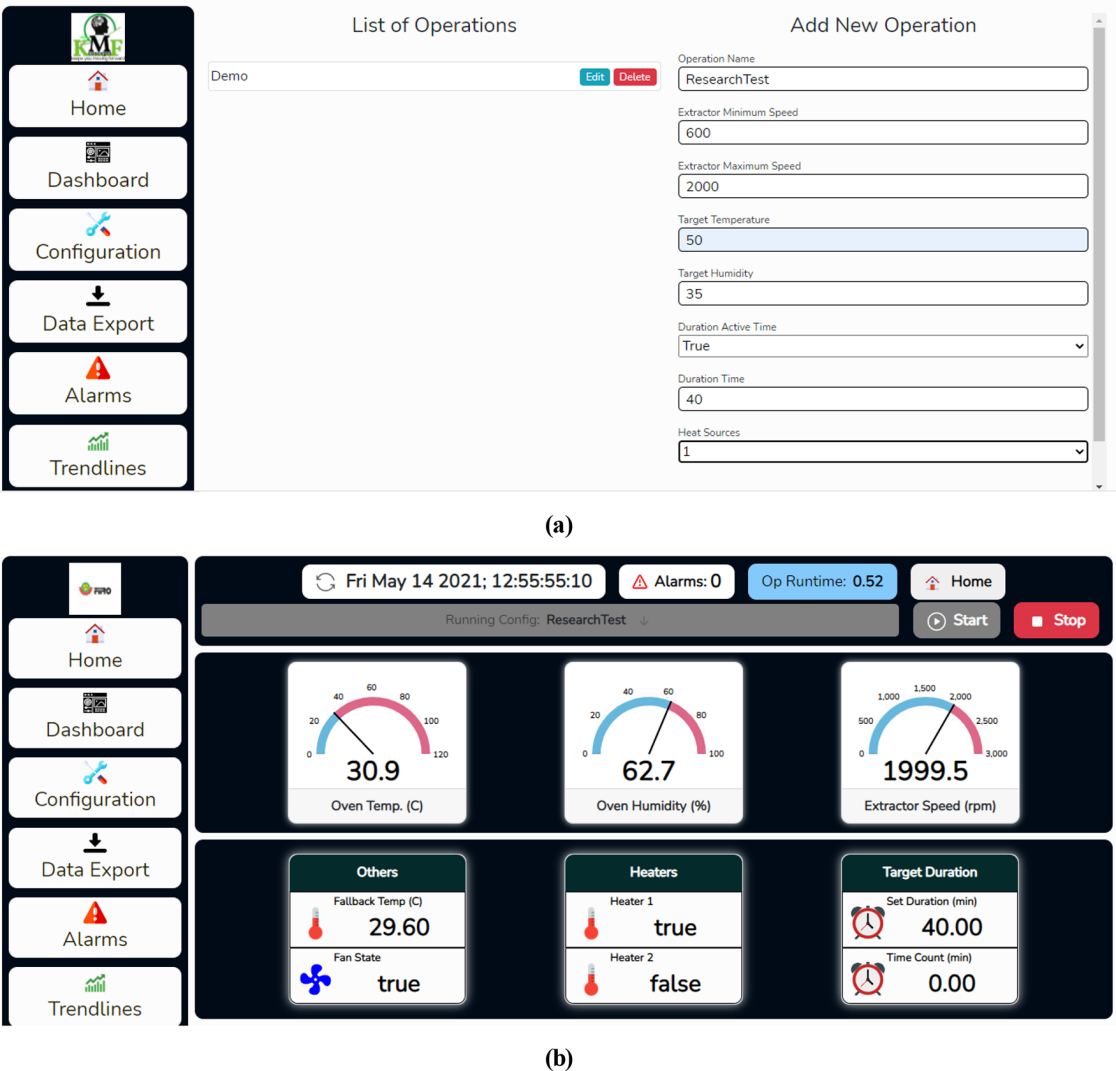
(a) Creating an operation profile on the dehydrator web app. (b) Dashboard page.

The operation profile named ResearchTest was created from the configuration page. Clicking on the configuration tab on the left side of the web page opens the user to the configuration page shown in Figure 13(a). The user can edit or delete existing operation profiles and create a new operation profile from this page. The target temperature was set to 50°C; the target humidity was set to 35%; the maximum and minimum fan speed was set to 2000rpm and 600rmp respective. Also, in the configuration, duration time was activated, and the desired duration time for the operation was set to 40 minutes. The newly created operation was selected on the dashboard page shown in Figure 13(b), and the start button was clicked for the operation to commence.

The user can view real-time operating parameters such as the drying chamber temperature and humidity, and fan speed on the dashboard page. Furthermore, other operation details such as set time for the operation and operation timer are shown on the dashboard page. The user can navigate to other pages by clicking on different tabs on the web page's left side.

**Figure 14.**
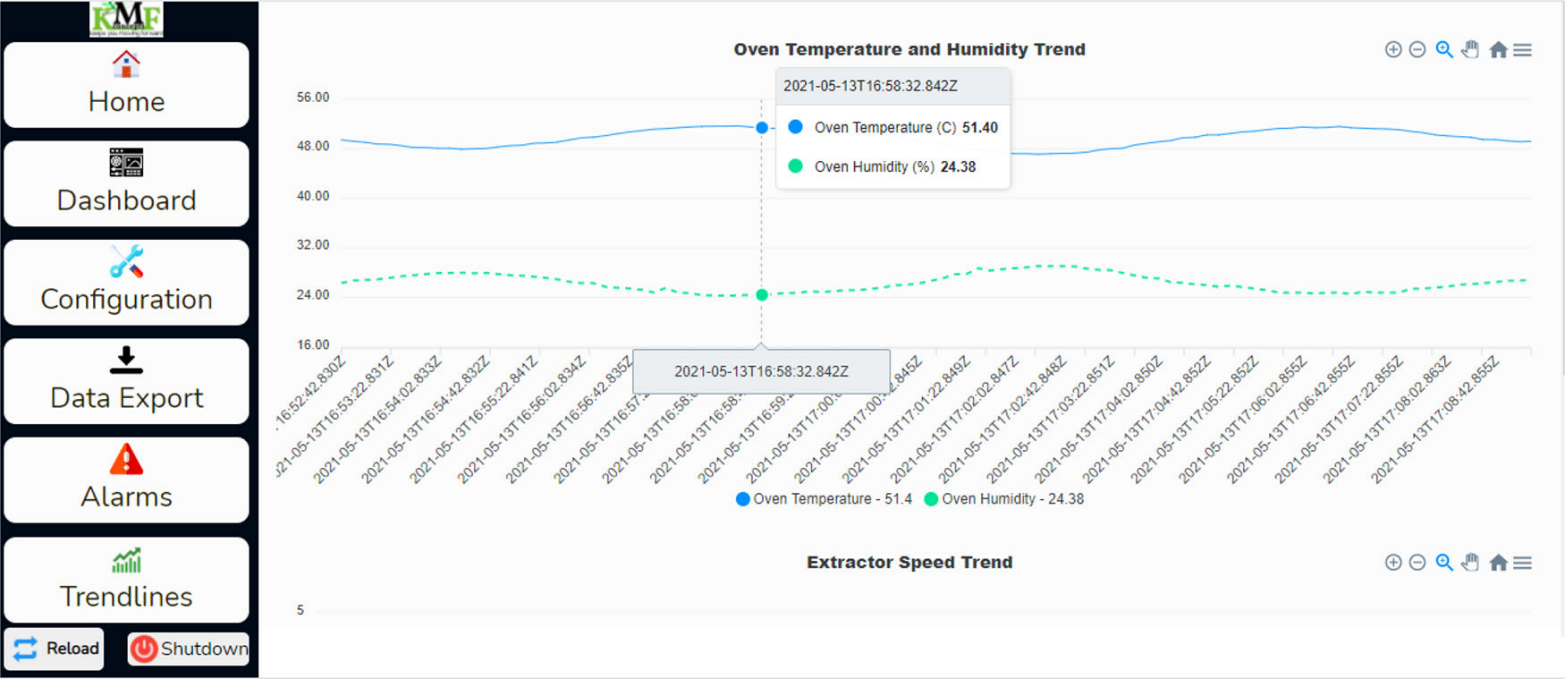
Screenshot chart on the dehydrator web page.

**Figure 15.**
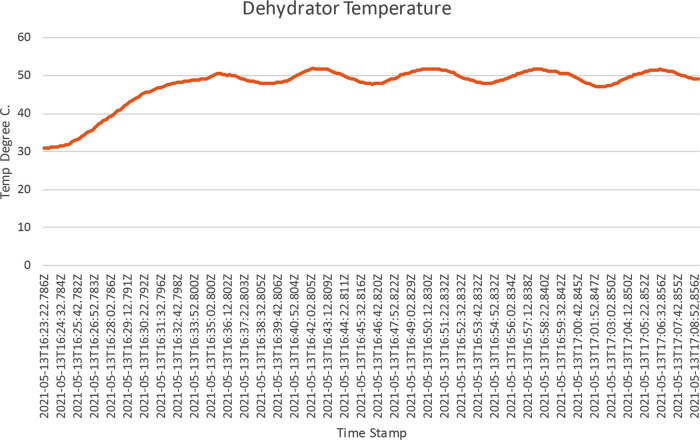
Chart of ResearchTest operation in Microsoft Office 365 Excel. The chart shows temperature in °C as a function of time for the Operational Test on PID Temperature Control.

The trendlines page can view the operation trends, which can be viewed by clicking on the Trendlines tab on the left side of the webpage. The trend chart for this test operation is shown in Figure 14. This shows the temperature and the humidity chart; as shown in the chart, the drying chamber temperature recorded was between 48.5°C to 51°C, around 50°C set as the target temperature. The raw data for this operation was downloaded from the dehydrator web interface and further analysed, as shown in Figure 15. The temperature was around 50°C, as shown on the trend charts. The data is also available in Excel format. ^31^

Furthermore, the data operation data was downloaded from the download page; navigation to this page was done by clicking on the Data Export tab. The data was downloaded in excel format and was further processed in excel to produce Figure 15, which shows how the PID controller forces the drying chamber temperature around 50°C, the set temperature.

## Discussion

Based on the charts above, during the ResearchTest operation, the heater raised the system temperature to the target temperature and stayed around it. The PID control is not as perfect as expected because the process temperature toggled between 48.7°C to 51.8°C. This is because the test was carried out in open space and not in the closed drying chamber that the oven will provide after fabrication. The temperature PID control performance can be improved on the final implementation by tuning the PID controller parameters to reduce the ripple. Furthermore, the frontend section of the web application was tested to ensure that it was in sync with the backend section that interacts with the system hardware and manages the database. The dehydrator has been designed but has not been fabricated. So only an operational test could be performed to simulate how the system would perform in an enclosed chamber. Future work would involve creating a physical prototype of the design carried out in this work and validating the operational test.

## Conclusion

The setup discussed in this work showed a design for a dehydrator or oven with full control over the drying chamber temperature and humidity. Gas is used as a source of heat energy because it is readily available and suitable for continuous industrial operation. Also, the setup gives the user the flexibility to create different operating profiles for different crops to make the dehydrator suitable for drying any type of crops or food samples. The user access to the historical operation data will provide insight into creating a better operation profile for a particular crop of interest.

Furthermore, the hybrid power solution provided gives the user the opportunity to use the dehydrator in remote places that are not connected to the national grid or places that experience unstable power supply.

## Data availability

### Underlying data

Zenodo. ResearchTest Data. https://doi.org/10.5281/zenodo.5105996. ^31^

This project contains the following underlying data:

- ResearchTest data.xlsx. (Temperature in °C as a function of time for the Operational Test on PID Temperature Control)

Data are available under the terms of the Creative Commons Attribution 4.0 International license (CC-BY 4.0).

## Software availability

Software available from: https://zenodo.org/record/5126962. ^32^

Source code available from: https://github.com/vickkiee/Dehydrator_Software.

Archived source code at time of publication: Dehydrator Software, https://doi.org/10.5281/zenodo.5126962.

License: Creative Commons Attribution 4.0 International (CC-BY 4.0).
