# Supplementary material for: Conceptual design of smart multi-farm produce dehydrator using a low-cost programmable logic controller and raspberry pi
Source: F1000Res. 2021 Nov 11;10:810. Originally published 2021 Aug 16. [Version 2] doi: 10.12688/f1000research.54463.2 (PMC8609401; doi:10.12688/f1000research.54463.2)
Supplement: Peer review history

# Peer review: Reviewer response for version 2

**DOI:** 10.5256/f1000research.79182.r99907

**Published:** 2021-11-22

**Authors:** Henry Inegbedion

**Affiliations:** 1 Department of Business Studies, Landmark University, Ilorin, Nigeria

The authors have included a section on the financial feasibility of the dehydrator. The section emphasises the cost implication of using the dehydrator without indicating the projected revenues that will permit its feasibility. The financial feasibility of the equipment will only be defined if the projected costs and revenues are presented and the profitability defined.

 The authors should make sure that they include the projected revenues and indicate the profitability of the equipment unfailingly.

Is the rationale for developing the new method (or application) clearly explained?

Yes

Is the description of the method technically sound?

Yes

Are the conclusions about the method and its performance adequately supported by the findings presented in the article?

Yes

If any results are presented, are all the source data underlying the results available to ensure full reproducibility?

Partly

Are sufficient details provided to allow replication of the method development and its use by others?

Partly

Reviewer Expertise:

Economics, Finance, Operations Research, Marketing and Agricultural Science

I confirm that I have read this submission and believe that I have an appropriate level of expertise to confirm that it is of an acceptable scientific standard.

# Peer review: Reviewer response for version 2

**DOI:** 10.5256/f1000research.79182.r99908

**Published:** 2021-11-15

**Authors:** Marly Grajales Amorocho, Johan Sebastian Velez Giraldo

**Affiliations:** 1 Facultad de Ciencias Básicas y Tecnologías, Doctorado en Ciencias, Grupo de Investigación en Biodiversidad y Biotecnología de la Universidad del Quindío (GIBUQ), Universidad del Quindío, Armenia, Colombia; 2 Facultad de Ciencias Básicas y Tecnologías, Maestría en Ciencias Biología Vegetal, Grupo de Investigación en Biodiversidad y Biotecnología de la Universidad del Quindío (GIBUQ), Universidad del Quindío, Armenia, Colombia

Confirm our approved status.

Is the rationale for developing the new method (or application) clearly explained?

Yes

Is the description of the method technically sound?

Yes

Are the conclusions about the method and its performance adequately supported by the findings presented in the article?

Partly

If any results are presented, are all the source data underlying the results available to ensure full reproducibility?

Yes

Are sufficient details provided to allow replication of the method development and its use by others?

Yes

Reviewer Expertise:

Biology, agriculture, biotechnology.

We confirm that we have read this submission and believe that we have an appropriate level of expertise to confirm that it is of an acceptable scientific standard.

# Peer review: Reviewer response for version 1

**DOI:** 10.5256/f1000research.57952.r95420

**Published:** 2021-11-01

**Authors:** Marly Grajales Amorocho, Johan Sebastian Velez Giraldo

**Affiliations:** 1 Facultad de Ciencias Básicas y Tecnologías, Doctorado en Ciencias, Grupo de Investigación en Biodiversidad y Biotecnología de la Universidad del Quindío (GIBUQ), Universidad del Quindío, Armenia, Colombia; 2 Facultad de Ciencias Básicas y Tecnologías, Maestría en Ciencias Biología Vegetal, Grupo de Investigación en Biodiversidad y Biotecnología de la Universidad del Quindío (GIBUQ), Universidad del Quindío, Armenia, Colombia

We do not know if the focus of the article is in our area, since it is on agricultural engineering, on the design of the dehydrator and in our case it would be more appropriate to evaluate the purpose or the function of the equipment, However, we give our review:

 The article reports the design of a new equipment for drying agricultural products which can be used anywhere in the world, mainly in those regions where climatic conditions such as high rainfall and low solar radiation prevent moisture removal to a safe level for storage. In addition, the design of the equipment is thought to be easy to operate since it has an automated electronic system that will control the conditions necessary for the dehydration process to occur if the products lose their natural properties. The design is novel, inexpensive and facilitates a continuous and permanent, automated process.

 The conceptual design conforms to the proposed parameters, however they conclude as if it had been used and so far there is no evidence that it works. It has a very good application in agriculture but it is more an engineering design. However, we suggest rewriting the conclusion that is formulated as a justification.

Is the rationale for developing the new method (or application) clearly explained?

Yes

Is the description of the method technically sound?

Yes

Are the conclusions about the method and its performance adequately supported by the findings presented in the article?

Partly

If any results are presented, are all the source data underlying the results available to ensure full reproducibility?

Yes

Are sufficient details provided to allow replication of the method development and its use by others?

Yes

Reviewer Expertise:

Biology, agriculture, biotechnology.

We confirm that we have read this submission and believe that we have an appropriate level of expertise to confirm that it is of an acceptable scientific standard.

# Peer review: Reviewer response for version 1

**DOI:** 10.5256/f1000research.57952.r95419

**Published:** 2021-10-18

**Authors:** Henry Inegbedion

**Affiliations:** 1 Department of Business Studies, Landmark University, Ilorin, Nigeria

The authors investigated the “conceptual design of smart multi-farm produce dehydrator using a low-cost programmable logic controller and raspberry”. The research problem is very important. This is a clear case example of applied research as the authors seek to provide a solution to the age-long dilemma that has been confronting farmers in Sub-Saharan Africa since its inception. The results of the study promise to contribute to the stabilization of the farm products, which are often very cheap during harvest seasons because the farmers are often willing to part with the products due to the high degree of perishability.

 Nevertheless, I am persuaded that the paper has a gap, which the authors should bridge in order to enhance the quality of the paper. I suggest that they indicate the financial feasibility of the equipment. The authors should indicate the capacity of the dehydrator in terms of a specific farm produce. Based on this capacity, estimates of the expected benefits of the specific farm produce in post-harvest seasons should be forecasted. Taking cognizance of the cost of producing and/or purchasing the multi-farm produce dehydrator vis-à-vis the expected returns from the post-harvest sales of the preserved farm produce, the financial benefit of the equipment should be indicated.

 The above is important because the value of the dehydrator to a farmer is a function of its capacity to enhance their economic fortunes. While the government may, in addition, be interested in using the equipment to enhance food security, the possibility of using the equipment to generate revenue will stimulate their interest and thus attract their attention faster. The financial feasibility of the equipment will enhance the value of this article.

 Lastly, the authors should indicate whether this proposed equipment is the first of its kind. If not, they should indicate what is unique about this very one.

Is the rationale for developing the new method (or application) clearly explained?

Yes

Is the description of the method technically sound?

Yes

Are the conclusions about the method and its performance adequately supported by the findings presented in the article?

Yes

If any results are presented, are all the source data underlying the results available to ensure full reproducibility?

Partly

Are sufficient details provided to allow replication of the method development and its use by others?

Partly

Reviewer Expertise:

Economics, Finance, Operations Research, Marketing and Agricultural Science

I confirm that I have read this submission and believe that I have an appropriate level of expertise to confirm that it is of an acceptable scientific standard, however I have significant reservations, as outlined above.

# Response

**Published:** 2021-11-05

**Authors:** victoria Oguntosin

**Affiliations:** Covenant University, Nigeria

We thank you for your time in reviewing the article.

 To emphasize the uniqueness of this dehydrator, none of these designs in the reviewed literature have control of both the temperature and humidity of the drying chamber simultaneously. In this work, the designed dehydrator will ensure the even distribution of heat within the drying chamber and, at the same, regulate both the temperature and the humidity of the drying chamber. Furthermore, the dehydrator will be user-friendly, such that the user can create different operation profiles for different types of crops or any drying sample, and also set the desired duration for the operation.

 The above has been explained in the article.

 Also, the paper has been updated with a section on the financial feasibility of the dehydrator. This section has the bill of quantities of materials used in the development of the dehydrator and discusses the capacity of the equipment. The pricing strategy of the equipment was also discussed for low-income farmers since the device is intended to be used in low-income countries.
